# Effect of Dietary Crude Protein and Apparent Metabolizable Energy Levels on Growth Performance, Nitrogen Utilization, Serum Parameter, Protein Synthesis, and Amino Acid Metabolism of 1- to 10-Day-Old Male Broilers

**DOI:** 10.3390/ijms25137431

**Published:** 2024-07-06

**Authors:** Yao Yu, Chunxiao Ai, Caiwei Luo, Jianmin Yuan

**Affiliations:** State Key Laboratory of Animal Nutrition and Feeding, College of Animal Science and Technology, China Agricultural University, Beijing 100193, China; 18611146962@163.com (Y.Y.); 2018304010108@cau.edu.cn (C.A.); 17330939818@163.com (C.L.)

**Keywords:** crude protein, metabolizable energy, growth performance, amino acid metabolism

## Abstract

This research compared how different levels of dietary crude protein (CP) and apparent metabolizable energy (AME) affect the growth performance, nitrogen utilization, serum parameters, protein synthesis, and amino acid (AA) metabolism in broilers aged 1 to 10 days. In a 4 × 3 factorial experimental design, the broilers were fed four levels of dietary CP (20%, 21%, 22%, and 23%) and three levels of dietary AME (2800 kcal/kg, 2900 kcal/kg, and 3000 kcal/kg). A total of 936 one-day-old male Arbor Acres broilers were randomly allocated to 12 treatments with 6 replications each. Growth performance, nitrogen utilization, serum parameter, gene expression of protein synthesis, and AA metabolism were evaluated at 10 d. The results revealed no interaction between dietary CP and AME levels on growth performance (*p* > 0.05). However, 22% and 23% CP enhanced body weight gain (BWG), the feed conversion ratio (FCR), total CP intake, and body protein deposition but had a detrimental effect on the protein efficiency ratio (PER) compared to 20% or 21% CP (*p* < 0.05). Broilers fed diets with 2800 kcal/kg AME showed increased feed intake (FI) and inferior PER (*p* < 0.05). Broilers fed diets with 3000 kcal/kg AME showed decreased muscle mRNA expression of mammalian target of the rapamycin (mTOR) and Atrogin-1 compared to those fed diets with 2800 kcal/kg and 2900 kcal/kg AME (*p* < 0.05). Increasing dietary CP level from 20% to 23% decreased muscle mTOR and increased S6K1 mRNA expression, respectively (*p* < 0.05). The muscle mRNA expression of Atrogin-1 was highest for broilers fed 23% CP diets (*p* < 0.05). The mRNA expression of betaine homocysteine methyltransferase (BHMT) and Liver alanine aminotransferase of the 22% and 23% CP groups were higher than those of 20% CP (*p* < 0.05). Significant interactions between dietary CP and AME levels were observed for muscle AMPK and liver lysine-ketoglutarate reductase (LKR) and branched-chain alpha-keto acid dehydrogenase (BCKDH) mRNA expression (*p* < 0.05). Dietary AME level had no effect on muscle AMPK mRNA expression for broilers fed 21% and 22% CP diets (*p* > 0.05), whereas increasing dietary AME levels decreased AMPK mRNA expression for broilers fed 23% CP diets (*p* < 0.05). The mRNA expression of LKR and BCKDH was highest for broilers fed the diet with 2800 kcal/kg AME and 22% CP, while it was lowest for broilers fed the diet with 3000 kcal/kg AME and 20% CP. The findings suggest that inadequate energy density hindered AA utilization for protein synthesis, leading to increased AA catabolism for broilers aged 1 to 10 days, and a dietary CP level of 22% and an AME level of 2900 to 3000 kcal/kg may be recommended based on performance and dietary protein utilization.

## 1. Introduction

Dietary crude protein (CP) and energy are fundamental nutrients in animal production. Early in 1994, the National Research Council (NRC) recommended specific CP and metabolizable energy (ME) requirements for broilers aged 1–21 days and 22–42 days, respectively [1]. In recent years, there has been a growing focus on nutrient needs during the starter phase. This is because of the rapid protein synthesis during this phase and the significant link between the weight of the starter phase and final body weight (BW) in production [2]. Researchers have recommended a dietary CP level of 23%, 21.23%, or 21% [3,4,5] and a dietary ME level of 2900 kcal/kg or 3000 kcal/kg for starter broilers [3,6]. Breeding companies recommend lower-energy diets. Arbor Acres nutrient specifications (2022) suggest 22% CP and 2975 kcal/kg ME for 1–10-day-old broilers [7], while Cobb nutrient specifications (2019) suggest 21–22% CP and 2900 kcal/kg nitrogen-corrected apparent metabolizable energy (AMEn) for 1–12-day-old broilers [8].

Dietary protein is essential for protein synthesis in animals, while ME supplies ATP for this process [9]. Therefore, the appropriate balance of dietary CP and ME is crucial for body protein synthesis. Low protein combined with high-ME diets can hinder growth performance because high ME can limit feed intake, resulting in insufficient protein intake for protein synthesis [10]. However, a high level of protein and a low-ME diet may lead to an inadequate ME supply for growth and would accelerate amino acids (AAs) catabolized into ammonia or uric acid (UA) [11,12].

Studies have shown that the mammalian target of the rapamycin (mTOR) pathway is essential in regulating muscle protein synthesis [13,14]. It is widely acknowledged that AAs are necessary to activate this pathway [15]. In addition to AAs, muscle fiber protein synthesis also relies on adequate energy. When cellular energy levels are low, AMP-activated protein kinase (AMPK) is activated, which serves as a crucial energy sensor and inhibits mTOR activity [16]. Additionally, researchers found that mRNA expression of AA oxidation enzymes, like ornithine acetyltransferase, lysine-ketoglutarate reductase (LKR), and branched-chain alpha-keto acid dehydrogenase (BCKDH), increased during energy restriction [17]. This suggests that increased energy preserves AAs from breakdown, allowing them to be used for protein synthesis. However, the impact of dietary energy density on protein synthesis and AA utilization in broilers remains uncertain.

Currently, there is a trend toward recommending lower-protein diets due to the growing demand for protein sources. Broiler breeder companies often suggest lower ME values. Therefore, providing a balanced ME and CP is crucial to achieving optimal performance and CP utilization. This study aimed to assess the effects of dietary CP and apparent metabolizable energy (AME) levels on growth performance, body composition, protein synthesis, and AA metabolism to determine the most suitable AME and CP levels for optimizing production and protein utilization in young broilers (1–10 days).

## 2. Results

### 2.1. Growth Performance

There was no significant interaction of dietary CP and AME levels on performance parameters (*p* > 0.05) (Table 1). Broilers given diets with 22% and 23% CP showed the highest BW and body weight gain (BWG), significantly surpassing those on a 21% CP diet. Conversely, broilers on a 20% CP diet exhibited the lowest BW and BWG. Increasing dietary CP levels from 20% to 22% increased feed intake (FI), but a further increase to 23% reduced FI (*p* < 0.05). The feed conversion ratio (FCR) improved as dietary CP levels increased (*p* < 0.05). However, dietary AME levels did not significantly affect BW and FCR (*p* > 0.05). Nonetheless, broilers fed diets with 2800 kcal/kg AME exhibited higher FI (*p* < 0.05). The mortality was not different, and the overall mortality rate was an acceptable 1.07%.

### 2.2. Body Composition of Broilers

There was no significant interaction of dietary CP and AME levels on body composition (*p* > 0.05) (Table 2). Broilers fed diets with 20% CP had the lowest body water content (*p* < 0.05). Additionally, the body protein content of broilers fed diets with 23% CP was not significantly different from that of those fed 22% CP diets, but it was higher than that of broilers fed diets with 20% and 21% CP (*p* < 0.05). Body fat content decreased with increasing dietary CP levels (*p* < 0.05). Broilers fed diets with 3000 kcal/kg AME had lower body water content and higher body fat content compared to those fed diets with 2800 kcal/kg and 2900 kcal/kg AME (*p* < 0.05).

### 2.3. Nutrient Intake, Deposition and Efficiency

There was a significant interaction between dietary CP and AME levels on body fat deposition (*p* < 0.05) (Table 3). Increasing dietary AME levels increased body fat deposition of broilers fed 21% and 22% CP diets, but dietary AME levels did not affect the body fat deposition of broilers fed 20% and 23% CP diets (*p* > 0.05). Total CP intake increased with dietary CP levels (*p* < 0.05). Additionally, the total CP intake of broilers fed diets with 2800 kcal/kg AME was higher than that of those fed diets with 2900 or 3000 kcal/kg AME (*p* < 0.05). There was no significant difference between 22% and 23% CP diets for total AME intake, but there was for higher than 20% and 21% CP diets (*p* < 0.05). Body protein deposition was highest for 22% and 23% CP diets, whereas it was lowest for 20% CP diets (*p* < 0.05). The protein efficiency ratio (PER) of broilers fed diets with 21% and 20% CP was similar but superior to that of diets with 22% and 23% CP (*p* < 0.05). Increasing dietary AME from 2800 kcal/kg to 2900 or 3000 kcal/kg improved PER (*p* < 0.05). Broilers fed diets with 20% CP had an inferior energy efficiency ratio (EER) compared to those fed diets with 21% to 23% CP. Moreover, increasing dietary AME from 2800 or 2900 kcal/kg to 3000 kcal/kg had a detrimental effect on the EER (*p* < 0.05).

### 2.4. Excreta Nitrogen Content and Nitrogen Retention

There were significant interactions between dietary CP and AME levels on excreta nitrogen (N) content and N retention efficiency (*p* < 0.05) (Table 4). The N content was highest for broilers fed the 23% CP and 3000 kcal/kg AME diet, and it was lowest for broilers fed the 20% CP and 2900 kcal/kg AME diet. Increasing dietary AME levels improved the N efficiency of broilers fed 20% CP diets, but dietary AME levels did not affect the N efficiency of broilers fed 21–23% CP diets (*p* > 0.05).

### 2.5. Serum Parameters

There were significant interactions between dietary CP and AME levels on serum UA and glucose (GLU) concentrations (*p* < 0.05) (Table 5). The serum UA concentration was highest for broilers fed the 23% CP and 2800 kcal/kg AME diet, and it was lowest for broilers fed the 20% CP and 2800 kcal/kg AME diet. Increasing dietary AME from 2800 kcal/kg to 3000 kcal/kg increased serum GLU concentration in broilers fed diets with 20% CP, whereas it had no effect on broilers fed diets with 21–23% CP (*p* < 0.05).

The serum albumin (ALB) concentration increased in broilers fed 23% CP diets compared to broilers fed 20–22% CP diets (*p* < 0.05). Broilers fed 20% CP diets had higher serum triglyceride (TG) concentrations (*p* < 0.05). The higher dietary AME levels increased serum TG levels (*p* < 0.05).

### 2.6. Gene Expressions of Protein Synthesis and Catabolism in Breast Muscle

There were significant interactions between dietary CP and AME levels on AMPK and muscle-specific ring finger 1 (MuRF1) mRNA expression (*p* < 0.05) (Table 6). The dietary AME level did not affect AMPK mRNA expression in broilers fed diets with 21% and 22% CP. However, increasing dietary AME levels decreased AMPK mRNA expression in broilers fed diets with 23% CP (*p* < 0.05). Increasing dietary AME levels upregulated MuRF1 mRNA expression for broilers fed 20% CP diets, the higher dietary AME levels downregulated MuRF1 mRNA expression for broilers fed 22% and 23% CP diets (*p* < 0.05). Increasing dietary CP level from 20% to 23% decreased mTOR and increased ribosomal protein S6 kinase beta-1 (S6K1) mRNA expression, respectively (*p* < 0.05). The mRNA expression of Atrogin-1 was upregulated for broilers fed 23% CP diets (*p* < 0.05). The broilers fed 3000 kcal/kg AME diets decreased the mRNA expression of mTOR and Atrogin-1, as compared to broilers provided with 2800 kcal/kg and 2900 kcal/kg AME diets (*p* < 0.05).

### 2.7. Gene Expressions of AA Catabolism and Transaminase Activity in Liver

There were significant interactions between dietary CP and AME levels on LKR and BCKDH (*p* < 0.05) (Table 7). The mRNA expression of LKR and BCKDH was highest for broilers fed the 2800 kcal/kg AME and 22% CP diet and was lowest for broilers fed the 3000 kcal/kg AME and 20% CP diet. The mRNA expression of betaine homocysteine methyltransferase (BHMT) was downregulated for broilers fed 20% CP diets than that of 21–23% CP diets (*p* < 0.05). The BHMT mRNA expression was upregulated in broilers given 2800 kcal/kg AME diets as compared with broilers fed 2900 kcal/kg and 3000 kcal/kg diets (*p* < 0.05). The alanine aminotransferase (ALT) activity increased with increasing dietary CP levels (*p* < 0.05). The dietary CP and AME levels did not affect aspartate aminotransferase (AST) activity (*p* > 0.05).

## 3. Discussion

Feeding chickens the right levels of CP and AME is crucial for maximizing growth. This study investigated the various CP and AME levels on performance and protein efficiency during the starter phase. We found that reducing dietary CP from 23% to 22% did not affect BW but decreasing it further to 21% or 20% negatively impacted BWG. Similarly, it was also reported that reducing the CP of the starter diet from 23.2% to 21% depressed the growth performance of 10-day-old broilers [18]. The notable impact of dietary CP content on bird performance in the starter phase can be attributed to the high AA requirements of newly hatched chicks, which are obligatory for rapid growth. In contrast, another group showed that reducing CP levels (21.23% vs. 23.78%) did not impact broiler performance [4]. This might be because the essential amino acid (EAA) content of the low-CP diet remained the same as the control diet in their study, while the EAA varied with dietary CP levels in our study. We observed that dietary AME levels did not affect BWG, but FI increased with reduced AME levels. Likewise, reducing dietary AME from 3000 kcal/kg to 2900 kcal/kg or from 3000 to 2925 kcal/kg did not influence BWG in the starter period [3,6]. These observations imply that a low-energy diet could potentially counteract the negative effects on bird performance by increasing FI moderately. In contrast, another study demonstrated that increased FI could not compensate for poor BWG due to the physical constraints of feed consumption [19]. FCR was highly sensitive to the changes in dietary AME and CP density, and we noticed a better FCR with increasing CP levels, while AME levels had no impact on FCR. Similarly, decreasing CP levels by 3% adversely affected FCR, with no significant impact of ME level on FCR during the initial 10 days [3]. Other research noted that reducing AME by 75 kcal/kg did not affect the FCR of 7-day-old broilers [6]. This inconsistency might be due to the age and AME levels across studies. To maximize broiler growth in the first 10 days, a CP level of 22% and an AME level of 2900 or 3000 kcal/kg are recommended.

Dietary CP levels have been widely shown to affect body composition [20,21]. In our study, we also found that low-CP diets significantly reduced body water and protein content. It was reported that reducing CP levels increased body fat content [22]. This can be attributed to the reduced protein synthesis on a low-CP diet, converting excess energy into fat accumulation. Higher dietary energy increases body fat content, and abdominal fat increases with dietary energy levels [23].

Serum ALB, primarily synthesized in the liver, reflects the liver protein synthesis function. In our study, the higher ALB content in 23% CP diets may be due to the improved availability of AA for protein synthesis. UA is the major end product of nitrogen catabolism in birds, and it manifests the direction of AA metabolism [24]. Studies have shown that serum UA content was positively correlated with dietary CP levels, whereas it was negatively correlated with dietary energy density [25]. Similarly, we found that the highest-CP and lowest-AME diet had the highest UA content, indicating an imbalance of CP and AME, which is detrimental to AA utilization. TG is primarily stored in adipose tissue, and it reflects lipid metabolism balance. The lower serum TG content in high-CP diets indicates less lipid deposition in bird tissue. This aligns with the finding that increasing CP levels reduced the expression of genes associated with lipogenesis [26].

In our study, dietary nutrients significantly affected the bird growth rate and body protein deposition. AA enhances muscle protein synthesis mainly through the activation of mTOR and its downstream protein S6K1 [27]. A low-protein diet would inhibit mTOR and S6K1 activity [28]. Similarly, our study revealed that S6K1 mRNA expression upregulated dietary CP levels, consistent with higher body protein deposition in broilers fed 22% and 23% CP diets. This suggests that dietary CP levels should be at least 22% to maximize body protein synthesis. Despite the increased protein deposition, higher dietary CP levels could reduce protein retention efficiency [29,30]. In this study, an inferior PER was noted as dietary CP levels increased. Consuming high-CP diets may cause some AA to exceed broiler requirements. This can lead to increased uptake and breakdown of these AAs by the liver, which helps in AA metabolism. The ALT activity and BHMT mRNA expression increased with dietary CP levels, indicating accelerated AA catabolism with higher dietary CP levels. Higher CP levels increased Atrogin-1 mRNA expression in breast muscle in this study. Atrogin-1 and MuRF1 are two muscle-specific E3 ubiquitin ligases, which play a role in tissue protein degradation [31]. This indicates that high dietary CP accelerates the protein turnover of breast muscle.

Muscle protein deposition relies on both AA and AME for ribosomal protein synthesis [9]. Therefore, a limitation in dietary energy would hinder protein synthesis. In the present study, body protein deposition was not affected by dietary AME levels. This could be because broilers fed low-energy diets increase their FI to meet their energy needs. However, when fed low-energy diets, total protein intake also increases, resulting in AA intake surpassing the need for protein synthesis, which potentially increases the hepatic uptake and catabolism of those AA. The literature indicates that proteins or AAs would catabolize to provide energy when AAs are excessive or imbalanced, or when there is a shortage of fat and carbohydrates [32]. The mRNA expression of LKR and BCKDH increased with reducing dietary AME for broilers fed 22% CP diets, and reducing dietary AME from 3000 kcal to 2800 kcal/kg AME increased the hepatic mRNA expression of BHMT. Similarly, it was found that ornithine acetyltransferase, BCKDH, and LKR increased during energy restriction [17], which reduces carbohydrate oxidation and shifts to AA catabolism in order to save carbohydrates during energy restriction. In this study, Atrogin-1 mRNA expression increased with lower AME levels, suggesting that dietary energy deficiency also accelerates protein breakdown. The increased AA oxidation and protein hydrolysis of broilers fed 2800 kcal/kg AME diets corresponded with their inferior PER. Similarly, another study noted a deterioration in dietary protein utilization efficiency with reduced dietary AME levels [33]. In addition, it was observed that a 20% reduction in ME supply decreased N retention [34]. Therefore, ensuring adequate energy content is crucial to maximizing protein retention efficiency.

## 4. Materials and Methods

### 4.1. Experimental Design and Bird Management

All animal procedures received approval from the Animal Ethical Committee of China Agricultural University (Protocol Number: AW40703202-1-5). A total of 936 one-day-old male Arbor Acres broilers were allocated to environment-controlled chambers and randomly divided into 12 treatments with 6 replications and 13 birds per cage. A 3 × 4 factorial experimental design was adopted to provide broilers with 3 levels of dietary AME (2800, 2900, and 3000 kcal/kg) and 4 levels of dietary CP (20%, 21%, 22%, and 23%). Before formulating the diets, corn, soybean meal, and corn gluten meal were analyzed for AME, CP, and AA density using near-infrared spectroscopy. The ingredient composition and nutrient content of the diets are shown in Table 8. Titanium dioxide (TiO_2_) was used as a dietary marker at 5 g/kg in all diets to determine nutrient digestibility.

The birds were managed under the guidance of the Arbor Acres broiler management handbook. Broilers were fed pelleted diets ad libitum and had free access to water via nipple drinkers.

### 4.2. Productive Performance and Sample Collection

At d 10, BWG, FI, and FCR were determined. The PER was calculated as the protein intake per gram of weight gain and the EER was calculated as the AME intake divided by weight gain. On day 10, one bird from each replicate of dietary groups was selected and euthanized by electrocution. Blood was collected via the jugular vein and separated for serum at 4000 rpm and 4 °C for 15 min. The breast muscle and liver were isolated for gene expression and enzyme activity analysis. Another bird was euthanized and the whole bird was ground for the analysis of body composition, protein deposition, and fat deposition.

### 4.3. Nutrient Digestibility

The excreta of each replicate were collected between days 7 and 9 to calculate the N retention efficiency and AME of diets. The excreta collected from each replicate were dried in an oven at 65 °C and ground finely for further analysis.

### 4.4. Serum and Liver Biochemical Analyses

Serum GLU, TG, ALB, and UA were analyzed using an automatic biochemical analyzer. Liver ALT and AST activities were determined with a spectrophotometer using commercial kits (Beijing Solarbio Science & Technology Co., Ltd., Beijing, China).

### 4.5. Quantitative Real-Time Polymerase Chain Reaction Analysis

The procedures of RNA extraction and RT-PCR analysis were performed according to Zhang et al. (2022) [35]. The sequences for primers were obtained from other publications [36,37,38,39]. The primer sequences of mTOR, S6K1, Atrogin-1, MuRF1, and AMPK in the breast muscle and LKR, BHMT, and BCKDH in the liver are shown in Table 9. Genes were normalized to the relative expression of β-actin using the 2^−ΔΔCT^ method.

### 4.6. Chemical Analysis

Chemical analysis of samples was conducted according to AOAC (2023) [40]. Dry matter was determined by drying to a constant weight in an oven at 105 °C. The total N contents of diets, excreta, and ground whole-bird samples were analyzed using the standard Kjeldahl method. The gross energy of diets and excreta was analyzed using an automatic adiabatic calorimeter. The crude fat content of grounded whole-bird samples was analyzed using the Soxhlet extraction method.

### 4.7. Calculation and Statistical Analysis

The total tract nitrogen retention efficiency and AME of diets were calculated with the following formulas:

N retention efficiency (%) = [1 − (N_excreta_ × M_diet_)/(N_diet_ × M_excreta_)] × 100

AME (kcal/kg) = [1 − (GE_excreta_ × M_diet_)/(GE_diet_ × M_excreta_)] × GE_diet_

where N_diet_ is the N concentration in the diet, N_excreta_ is the N concentration in excreta, M_diet_ is the TiO_2_ concentration in the diet, M_excreta_ is the TiO_2_ concentration in excreta, GE_excreta_ is the GE of excreta, and GE_diet_ is GE of the diet.

Prior to the analysis of variance, Shapiro–Wilk’s test was performed to check the normality and Levene’s test was conducted for homogeneity of variance. The data were analyzed by a two-way ANOVA using the GLM procedure of SAS 9.0 (SAS Inst. Inc., Cary, NC, USA) to determine the main effects of dietary protein, energy, and their interaction. When a significant interaction was noted, a one-way ANOVA analysis was conducted, and Tukey’s test was applied to separate the means. A statistical significance level of *p* < 0.05 was observed.

## 5. Conclusions

It was concluded that the ideal levels for optimal growth performance are 22% for CP and 2900 to 3000 kcal/kg for AME. Increasing dietary CP levels deteriorated the N retention efficiency, and inadequate energy density hindered AA utilization for protein synthesis, leading to increased AA catabolism.

## Figures and Tables

**Table 1:** Effect of dietary CP and AME levels on growth performance of 10-day-old broilers.

CP, %	AME, kcal/kg	BW, g	BWG, g	FI, g	FCR
20	2800	247.3	201.7	288.3	1.436
	2900	245.0	200.2	282.2	1.401
	3000	249.7	204.5	268.2	1.399
21	2800	264.8	219.0	292.7	1.350
	2900	259.4	214.0	289.8	1.352
	3000	271.3	225.7	294.4	1.328
22	2800	274.3	228.7	305.7	1.348
	2900	269.8	224.3	295.8	1.334
	3000	271.2	225.3	295.5	1.336
23	2800	269.7	224.0	295.5	1.319
	2900	274.7	229.3	293.8	1.289
	3000	247.3	201.7	288.3	1.436
SEM		3.631	3.602	3.319	0.017
Main effect					
CP	20	247.5 ^c^	202.2 ^c^	280.2 ^c^	1.416 ^a^
	21	265.5 ^b^	219.9 ^b^	292.3 ^b^	1.343 ^b^
	22	271.8 ^a^	226.1 ^a^	299.0 ^a^	1.339 ^b^
	23	272.4 ^a^	227.0 ^a^	292.5 ^b^	1.299 ^c^
					
AME	2800	264.0	218.3	295.5 ^a^	1.363
	2900	263.1	217.9	290.4 ^b^	1.344
	3000	266.3	220.8	287.0 ^b^	1.338
*p*-Value					
CP		<0.001	<0.001	<0.001	<0.001
AME		0.297	0.325	0.001	0.156
CP × AME		0.494	0.480	0.054	0.937

CP: crude protein; AME: apparent metabolizable energy; BW: body weight; BWG: body weight gain; FI: feed intake; FCR: feed conversion ratio; SEM: standard error of means. Mean values in the same column with different superscripts (a, b, and c) are significant at *p* < 0.05.

**Table 2:** Effect of dietary CP and AME levels on body composition of 10-day-old broilers.

CP, %	AME, kcal/kg	Water, %	Protein, %	Fat, %
20	2800	71.86	16.07	8.83
	2900	71.98	16.06	9.10
	3000	71.02	15.85	9.73
21	2800	72.91	16.20	7.58
	2900	72.72	16.10	8.53
	3000	72.30	16.05	8.44
22	2800	72.86	16.47	7.53
	2900	73.14	16.27	7.77
	3000	71.92	16.10	8.49
23	2800	72.96	16.68	7.14
	2900	72.86	16.48	7.30
	3000	72.53	16.30	7.75
SEM		0.266	0.190	0.248
Main effect				
CP	20	71.51 ^b^	15.96 ^b^	9.33 ^a^
	21	72.71 ^a^	16.13 ^b^	8.03 ^b^
	22	72.64 ^a^	16.28 ^ab^	7.96 ^b^
	23	72.78 ^a^	16.49 ^a^	7.40 ^c^
				
AME	2800	72.76 ^a^	16.39	7.62 ^b^
	2900	72.74 ^a^	16.24	8.07 ^b^
	3000	71.89 ^b^	16.08	8.63 ^a^
*p*-Value				
CP		<0.001	0.019	<0.001
AME		<0.001	0.146	<0.001
CP × AME		0.705	0.996	0.335

Mean values in the same column with different superscripts (a, b, and c) are significant at *p* < 0.05.

**Table 3:** Effect of dietary CP and AME on the nutrient intake, body deposition, and nutrient efficiency ratio of 1- to 10-day-old broilers.

CP, %	AME, kcal/kg	Nutrient Intake		Body Deposition		Nutrient Efficiency	
		CP, g	AME, kcal	Protein, g	Fat, g	PER ^1^, g: g	EER ^2^, kcal/g
20	2800	58.2	792	32.7	23.3 ^abc^	0.289	3.93
	2900	57.5	795	32.6	24.2 ^a^	0.286	3.94
	3000	57.9	799	31.3	24.3 ^a^	0.296	4.01
21	2800	63.4	810	35.4	21.6 ^def^	0.289	3.69
	2900	61.4	836	34.8	23.0 ^abcd^	0.283	3.94
	3000	62.4	866	35.9	23.9 ^ab^	0.276	3.75
22	2800	68.9	849	38.0	22.1 ^cdef^	0.310	3.72
	2900	65.0	851	36.3	21.4 ^ef^	0.290	3.80
	3000	65.8	874	36.1	24.5 ^a^	0.292	3.88
23	2800	72.6	850	37.5	20.4 ^f^	0.323	3.81
	2900	71.5	844	37.8	21.6 ^def^	0.312	3.68
	3000	70.4	879	36.6	22.0 ^bcdef^	0.315	3.93
SEM		0.819	10.8	0.629	0.363	0.003	0.035
Main effect							
CP	20	57.9 ^d^	795 ^c^	32.2 ^c^	23.9	0.290 ^bc^	3.96 ^a^
	21	62.4 ^c^	838 ^b^	35.3 ^b^	22.8	0.283 ^c^	3.79 ^b^
	22	66.5 ^b^	858 ^a^	36.7 ^a^	22.7	0.294 ^b^	3.80 ^b^
	23	71.5 ^a^	857 ^a^	37.3 ^a^	21.3	0.316 ^a^	3.81 ^b^
							
AME	2800	65.5 ^a^	824 ^b^	35.7	21.9	0.300 ^a^	3.79 ^b^
	2900	64.1 ^b^	833 ^b^	35.4	22.5	0.292 ^b^	3.82 ^b^
	3000	63.8 ^b^	853 ^a^	34.9	23.7	0.295 ^b^	3.91 ^a^
*p*-Value							
CP		<0.001	<0.001	<0.001	<0.001	<0.001	<0.001
AME		0.003	<0.001	0.135	<0.001	0.003	0.001
CP × AME		0.380	0.343	0.289	0.002	0.086	0.077

^1^ PER: protein efficiency ratio, calculated as protein intake divided by weight gain. ^2^ EER: energy efficiency ratio, calculated as total AME intake divided by weight gain. Mean values in the same column with different superscripts (a, b, c, d, e, and f) are significant at *p* < 0.05.

**Table 4:** Effect of dietary CP and AME on excreta N content and N retention of broilers.

CP, %	AME, kcal/kg	N Content, %	N Retention Efficiency, %
20	2800	4.02 ^cde^	65.0 ^b^
	2900	3.78 ^e^	68.0 ^ab^
	3000	3.87 ^de^	71.0 ^a^
21	2800	4.04 ^cde^	67.7 ^ab^
	2900	3.95 ^cde^	68.3 ^ab^
	3000	4.00 ^cde^	66.6 ^ab^
22	2800	4.25 ^bcd^	65.9 ^ab^
	2900	4.36 ^abc^	65.9 ^ab^
	3000	4.14 ^cde^	69.3 ^ab^
23	2800	4.38 ^abc^	69.1 ^ab^
	2900	4.71 ^ab^	65.5 ^ab^
	3000	4.72 ^a^	69.2 ^ab^
SEM		0.098	1.217
Main effect			
CP	20	3.89	68.1
	21	4.00	67.5
	22	4.25	67.1
	23	4.60	67.9
			
AME	2800	4.17	67.0
	2900	4.20	66.9
	3000	4.18	69.0
*p*-Value			
CP		<0.001	0.734
AME		0.937	0.025
CP × AME		0.046	0.021

Mean values in the same column with different superscripts (a, b, c, d, and e) are significant at *p* < 0.05.

**Table 5:** Effect of dietary CP and AME levels on serum parameters of 10-day-old broilers.

CP, %	AME, kcal/kg	UA μmol/L	ALB g/L	TG mmol/L	GLU mmol/L
20	2800	732.9 ^b^	13.6	2.49	16.3 ^b^
	2900	990.4 ^ab^	13.2	3.08	25.0 ^a^
	3000	906.4 ^ab^	14.6	3.59	26.1 ^a^
21	2800	781.2 ^b^	14.3	1.89	18.3 ^ab^
	2900	997.9 ^ab^	14.5	1.85	20.9 ^ab^
	3000	766.9 ^b^	14.4	2.12	19.6 ^ab^
22	2800	1082.2 ^ab^	14.2	1.50	20.2 ^ab^
	2900	865.9 ^ab^	14.4	2.04	20.4 ^ab^
	3000	881.3 ^ab^	13.9	2.14	22.7 ^ab^
23	2800	1178.7 ^a^	15.4	2.00	25.3 ^a^
	2900	1010.2 ^ab^	16.2	1.73	22.6 ^ab^
	3000	980.4 ^ab^	15.1	1.98	19.1 ^ab^
SEM		77.521	0.408	0.268	1.850
Main effect					
CP	20	885.0	13.8 ^b^	3.06 ^a^	22.5
	21	839.9	14.4 ^b^	1.95 ^b^	19.7
	22	946.7	14.2 ^b^	1.87 ^b^	21.0
	23	1059.1	15.6 ^a^	1.90 ^b^	22.6
					
AME	2800	952.9	14.4	1.99 ^b^	20.1
	2900	962.6	14.6	2.18 ^ab^	22.2
	3000	883.9	14.5	2.49 ^a^	22.0
*p*-Value					
CP		<0.001	<0.001	<0.001	0.209
AME		0.309	0.728	0.046	0.197
CP × AME		0.017	0.148	0.387	<0.001

UA: uric acid; ALB: albumin; TG: triglyceride; GLU: glucose. Mean values in the same column with different superscripts (a and b) are significant at *p* < 0.05.

**Table 6:** Effect of dietary CP and AME on breast muscle gene expressions.

CP, %	AME, kcal/kg	AMPK	mTOR	S6K1	Atrogin-1	MuRF1
20	2800	1.00 ^e^	1.00	1.00	1.00	1.00 ^bcd^
	2900	1.53 ^b^	1.10	1.18	1.25	1.11 ^abcd^
	3000	1.15 ^cde^	0.95	1.26	1.07	1.51 ^a^
21	2800	0.94 ^e^	0.99	1.50	1.27	0.95 ^cd^
	2900	1.14 ^de^	0.88	1.28	1.03	1.33 ^abcd^
	3000	1.08 ^de^	0.88	1.14	1.01	1.26 ^abcd^
22	2800	1.51 ^b^	0.97	1.62	1.12	1.45 ^ab^
	2900	1.49 ^bc^	1.03	1.67	1.26	1.32 ^abcd^
	3000	1.21 ^bcde^	0.58	1.65	1.07	0.90 ^d^
23	2800	1.96 ^a^	0.97	1.60	1.64	1.42 ^abc^
	2900	1.42 ^bcd^	0.94	2.01	1.38	1.47 ^ab^
	3000	1.65 ^b^	0.74	2.08	1.05	1.01 ^bcd^
SEM		0.03	0.064	0.160	0.123	0.146
Main effect						
CP	20	1.23	1.02 ^a^	1.15 ^b^	1.11 ^b^	1.19
	21	1.05	0.91 ^ab^	1.31 ^b^	1.08 ^b^	1.18
	22	1.40	0.89 ^b^	1.65 ^a^	1.15 ^b^	1.35
	23	1.68	0.88 ^b^	1.90 ^a^	1.36 ^a^	1.30
						
AME	2800	1.35	0.98 ^a^	1.43	1.24 ^a^	1.21
	2900	1.39	0.99 ^a^	1.54	1.23 ^a^	1.41
	3000	1.24	0.79 ^b^	1.53	1.05 ^b^	1.15
*p*-Value						
CP		<0.001	0.040	<0.001	0.050	0.754
AME		0.024	<0.001	0.567	0.046	0.352
CP × AME		<0.001	0.090	0.243	0.131	0.004

AMPK: AMP-activated protein kinase; mTOR: mammalian target of the rapamycin; S6K1: ribosomal protein S6 kinase beta-1; MuRF1: muscle-specific ring finger 1. Mean values in the same column with different superscripts (a, b, c, d, and e) are significant at *p* < 0.05.

**Table 7:** Effect of dietary CP and AME on liver gene expression and transaminase activity of 10-day-old broilers.

CP, %	AME, kcal/kg	LKR	BHMT	BCKDH	ALT, U/g prot	AST, U/g prot
20	2800	1.00 ^b^	1.00	1.00 ^bc^	0.213	4.24
	2900	1.17 ^b^	0.79	1.73 ^abc^	0.228	4.58
	3000	0.74 ^b^	0.53	0.77 ^c^	0.233	3.84
21	2800	2.28 ^b^	1.60	1.54 ^abc^	0.269	4.52
	2900	1.57 ^b^	0.98	1.34 ^abc^	0.261	4.75
	3000	1.69 ^b^	1.67	2.25 ^ab^	0.226	4.44
22	2800	4.85 ^a^	2.02	2.53 ^a^	0.267	4.37
	2900	2.24 ^b^	1.35	1.64 ^abc^	0.314	4.34
	3000	1.48 ^b^	1.21	1.37 ^abc^	0.297	4.55
23	2800	1.82 ^b^	1.59	1.01 ^bc^	0.340	4.35
	2900	1.54 ^b^	0.96	0.98 ^bc^	0.264	4.37
	3000	1.69 ^b^	1.64	1.67 ^abc^	0.247	4.41
SEM		0.521	0.205	0.293	0.029	0.233
Main effect						
CP	20	0.93	0.76 ^b^	1.17	0.225 ^b^	4.22
	21	1.86	1.39 ^a^	1.72	0.252 ^ab^	4.57
	22	2.74	1.54 ^a^	1.85	0.284 ^a^	4.43
	23	1.69	1.40 ^a^	1.19	0292 ^a^	4.38
						
AME	2800	2.59	1.57 ^a^	1.52	0.272	4.37
	2900	1.68	1.03 ^b^	1.42	0.265	4.51
	3000	1.32	1.24 ^b^	1.51	0.252	4.32
*p*-Value						
CP		<0.001	<0.001	0.010	0.021	0.339
AME		0.017	0.002	0.871	0.544	0.472
CP × AME		0.041	0.097	0.003	0.278	0.552

LKR: lysine-ketoglutarate reductase (LKR); BHMT: betaine homocysteine methyltransferase; BCKDH: branched-chain alpha-keto acid dehydrogenase; ALT: alanine aminotransferase; AST: aspartate aminotransferase. Mean values in the same column with different superscripts (a, b, and c) are significant at *p* < 0.05.

**Table 8:** Ingredient composition and nutrient content of the experimental diets (%, as-fed basis).

Ingredient, %	Diet 1	Diet 2	Diet 3	Diet 4	Diet 5	Diet 6	Diet 7	Diet 8	Diet 9	Diet 10	Diet 11	Diet 12
Corn	59.00	57.40	55.80	55.54	54.62	53.70	52.07	51.84	51.60	49.10	49.45	49.80
Soybean meal	35.00	35.35	35.70	37.84	37.03	36.23	40.67	38.71	36.75	43.10	40.15	37.20
Corn gluten meal	0.00	0.00	0.00	0.00	0.66	1.31	0.00	1.31	2.63	0.00	1.88	3.75
Soybean oil	1.45	2.73	4.00	1.85	2.89	3.93	2.26	3.06	3.86	2.60	3.20	3.80
Dicalcium phosphate	1.62	1.64	1.66	1.70	1.72	1.73	1.79	1.79	1.79	1.86	1.86	1.85
Sodium chloride	0.35	0.35	0.35	0.35	0.35	0.35	0.35	0.35	0.35	0.35	0.35	0.35
Limestone	1.30	1.25	1.20	1.30	1.28	1.25	1.30	1.30	1.31	1.30	1.33	1.35
Vitamin premix ^1^	0.03	0.03	0.03	0.03	0.03	0.03	0.03	0.03	0.03	0.03	0.03	0.03
Mineral premix ^2^	0.20	0.20	0.20	0.20	0.20	0.20	0.20	0.20	0.20	0.20	0.20	0.20
Choline chloride 50%	0.20	0.20	0.20	0.20	0.20	0.20	0.20	0.20	0.20	0.20	0.20	0.20
Antioxidant	0.02	0.02	0.02	0.02	0.02	0.02	0.02	0.02	0.02	0.02	0.02	0.02
L-Lysine HCL 98.5%	0.10	0.10	0.10	0.16	0.18	0.21	0.21	0.26	0.31	0.26	0.33	0.40
D-Methionine 98%	0.20	0.21	0.21	0.26	0.26	0.26	0.31	0.31	0.30	0.36	0.35	0.34
L-Threonine 98.5%	-	-	-	0.02	0.02	0.03	0.04	0.05	0.06	0.05	0.07	0.08
L-Isoleucine 90%	-	-	-	0.01	0.02	0.02	0.03	0.03	0.04	0.04	0.05	0.05
L-Arginine 98%	-	-	-	-	0.01	0.02	-	0.02	0.04	-	0.03	0.05
Phytase 10,000 U/g	0.03	0.03	0.03	0.03	0.03	0.03	0.03	0.03	0.03	0.03	0.03	0.03
Titanium dioxide	0.50	0.50	0.50	0.50	0.50	0.50	0.50	0.50	0.50	0.50	0.50	0.50
Calculated nutrient content												
AME, kcal/kg	2800	2900	3000	2800	2900	3000	2800	2900	3000	2800	2900	3000
Crude protein, %	20.00	20.00	20.00	21.00	21.00	21.00	22.00	22.00	22.00	23.00	23.00	23.00
Digestible Lys, %	1.10	1.10	1.10	1.20	1.20	1.20	1.31	1.31	1.31	1.40	1.40	1.40
Digestible Met + Cys, %	0.80	0.80	0.80	0.88	0.88	0.88	0.96	0.96	0.96	1.02	1.02	1.02
Digestible Thr, %	0.72	0.72	0.72	0.79	0.79	0.79	0.86	0.86	0.86	0.92	0.92	0.92
Calcium, %	0.96	0.96	0.96	0.96	0.96	0.96	0.96	0.96	0.96	0.96	0.96	0.96
Available P, %	0.48	0.48	0.48	0.48	0.48	0.48	0.48	0.48	0.48	0.48	0.48	0.48
Analyzed nutrient content												
CP, %	20.21	20.39	21.28	21.67	20.90	20.99	22.52	22.10	22.25	24.49	24.33	24.38
AME, kcal/kg	2745	2816	2937	2769	2848	2915	2778	2894	2958	2866	2871	3047

^1^ The vitamin premix provided (per kg of diets) the following: vitamin A, 15,000 IU; vitamin D3, 3600 IU; vitamin E, 30 IU; vitamin K3, 3.00 mg; vitamin B2, 9.60 mg; vitamin B12, 0.03 mg; biotin, 0.15 mg; folic acid, 1.50 mg; pantothenic acid, 13.80 mg; nicotinic acid, 45 mg. ^2^ The trace mineral premix provided (per kg of diets) the following: Cu, 16 mg; Zn, 110 mg; Fe, 80 mg; Mn, 120 mg; Se, 0.30 mg; I, 1.50 mg.

**Table 9:** Primer sequences of RT-PCR.

Gene	Forward Sequences (5′-3′)	Reverse Sequences (5′-3′)
mTOR	AGTGAGAGTGATGCGGAGAG	GAAACCTTGGACAGCGGG
S6K1	GGTGGAGTTTGGGGGCATTA	GAAGAACGGGTGAGCCTAA
Atrogin-1	CCAACAACCCAGAGACCTGT	GGAGCTTCACACGAACATGA
MuRF1	GCTGGTGGAGAACATCATCG	GCTGGTGGAGAACATCATCG
AMPK	ATCTGTCTCGCCCTCATCCT	CCACTTCGCTCTTCTTACACCTT
LKR	AACACCAGCCATGAAGGAAC	TGAACGGTGTTCAGCAAGAC
BHMT	AGAGATTGTGATTGGAGATGGG	TGTTCTACTGTTGCTTCGGG
BCKDH	ACCTCTTCTCCGATGTGTACCG	TCGTAGAGCTCCATGGGGTAAT
β-actin	GAGAAATTGTGCGTGACATCA	CCTGAACCTCTCATTGCCA

β-actin expression level was used as an internal control.

## Data Availability

Datasets supporting the results of this article are included within the article.
